# Callus cell, shoot and stem proliferation data from pineapple crown and banana inflorescence in vitro: Biochemical and antioxidant properties

**DOI:** 10.1016/j.dib.2016.11.097

**Published:** 2016-12-06

**Authors:** ABM Sharif Hossain, Musamma M. Uddin

**Affiliations:** a Department of Biology, Faculty of Science, University of Hail, Saudi Arabia; b Biotechnology Program, Biological Science, Faculty of Science, University of Malaya, Kuala Lumpur, Malaysia

## Abstract

The data article contains the experimental data and images on the callus cell, shoot and stem proliferation from pineapple crown slice and banana inflorescence in vitro. Investigated data are related to the research article “Effects of benzylaminopurine and naphthalene acetic acid on proliferation and shoot growth of pineapple (*Ananas comosus* L. Merr) in vitro” Alsaif et al. (2011) [1] and “Plantlet Production through Development of Competent Multiple Meristem Cultures from Male Inflorescence of Banana, *Musa acuminta* cv. ׳Pisang Mas׳(AA)” Wirakarnain et al. (2008) [2]. In the experimental data 1, physiological, (shoot weight, number length and stem proliferation) biochemical (total sugar and chlorophyll) and nutritional ((K^+^ and NO_3_^−^) data using BAP, MS medium and NAA growth regulators in pineapple have been explored. In the experimental data 2, physiological, (callus weight, shoot number and length) biochemical (total sugar, chlorophyll, total phenol, DPPH) and nutritional (K^+^ and NO_3_^−^) data employing BAP +IAA, MS medium and NAA growth regulators in banana have been exhibited. Overall quantitative measurement was observed by Spectrophotometer. In the experimental data, BAP was shown the best effective hormone for the both pineapple and banana explants regeneration.

**Specialization Table**

**Table:** 

**Subject area:** Biology.
**More specific subject area:** Plant cell and Tissue culture Biotechnology.
**Type of data:** Related to our previously published work but unpublished current data.
**How data was acquired:** Culture in Growth chamber, antioxidant activity, DPPH free radical measured by spectrophotometer, Biochemical and mineral content determination.
**Data format:** Raw, analyzed.
**Experimental Factors**: Single factor different plant hormone concentrations (BAP, IAA, NAA).
**Experimental features:** 5 replicates were used as CRD design.
**Data source location:** University of Malaya, Kuala Lumpur, Malaysia and Hail University, Hail city, Saudi Arabia.
**Data accessibility:** Data are presented in this article.

**Value of the Data**

• Data show the increased callus and shoot proliferation at the concentration of BAP in pineapple and BAP+IAA in Banana in vitro culture that would be an innovative data compared to other researchers.
• Data signify the studies of physiological, nutritional, biochemical and antioxidant activity in pineapple and banana explants in vitro culture.
• Investigated data are useful to the researchers working in the area of Biological Chemistry, Plant Biotechnology and Biochemistry.

## 1 Data

**Fig. 1:**
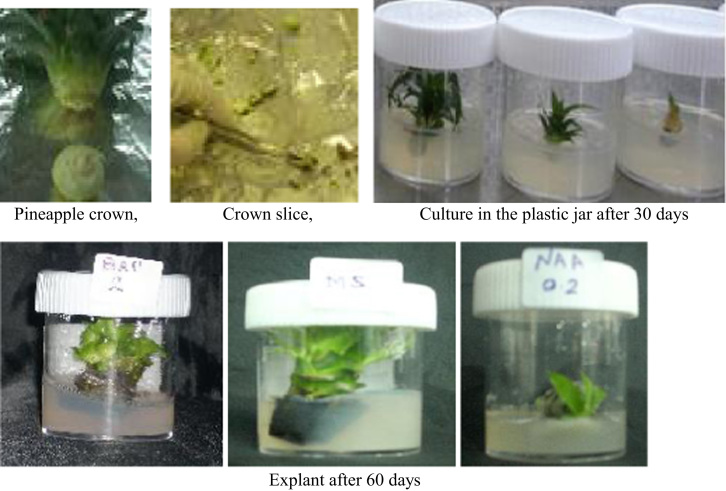
Photograph shows the culture from crown of pineapple and subculture from explants at different growth hormones.

**Fig. 2:**
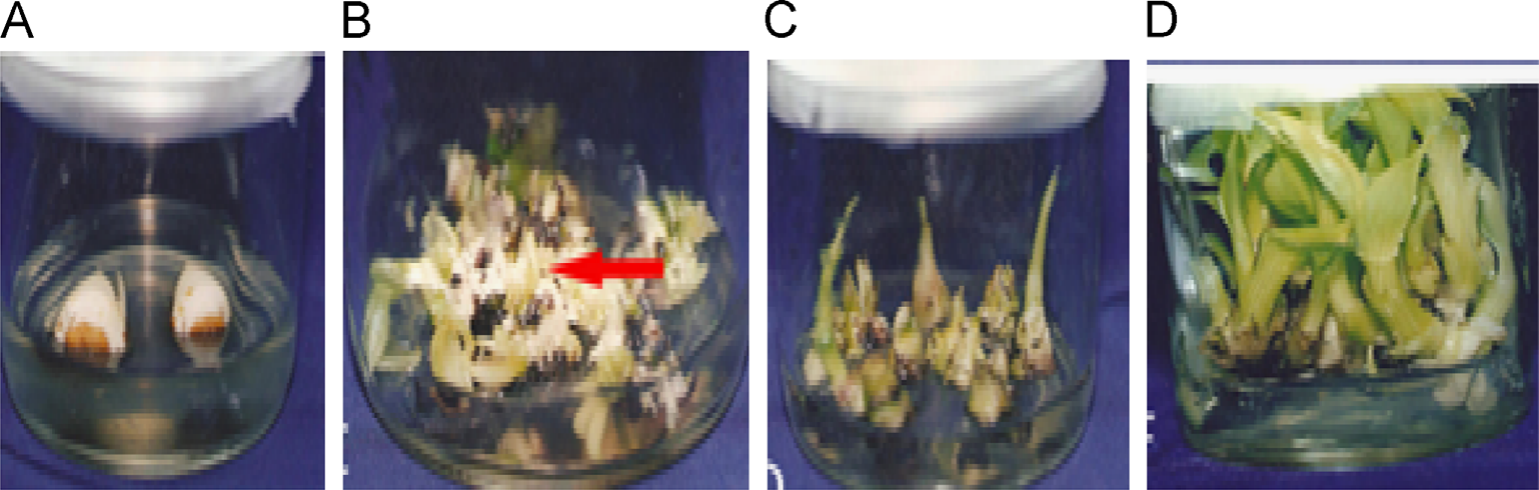
(A–E): Development of shoots from green scalps derived from male inflorescence of *Musa acuminta*: dissection of male bud was shortened to 6–8 cm. (B): The proliferation of white floral meristems cell and tissue. (C): Formation of shoots derived from white floral meristems cell and tissue. (D): Development of vigorous shoots derived from green competent meristem.

In the data, the effects of NAA and BAP on the shoot weight and stem length have been shown from pineapple explants (Table 1) [1]. In Table 2, total sugar, chlorophyll a, b and nutrient content determination have been exhibited from pineapple explants. Moreover, callus weight, shoot number and length have been shown from banana explants influenced by NAA and BAP+IAA hormones (Table 3) [2]. Table 4 has explored the total sugar, chlorophyll a, b, total phenol, DPPH activity and nutrient content from banana explants. In addition, Fig. 1 has represented the photograph of the culture from pineapple crown and subculture from explants. Fig. 2 shows the growth of shoot from male inflorescence of banana *(Musa acuminta).*

## 2 Experimental design, materials and methods

### 2.1 Experiment 1

#### 2.1.1 Medium preparation

MS medium [3] was prepared (1 L) from stock solutions and supplemented with sucrose at 30 g/l. The medium was adjusted to pH 5.7 before adding agar at 7.0 g L^−^^1^. The beaker containing the medium was placed over magnetic stirrer hot plate and heated to boiling to dissolve the agar and then dispensed equally (20 mL jar^−1^) into 24 glass jars (5×15 cm) with screw rim and plastic lid which were autoclavable. The medium was then autoclaved at 121 °C and 1.5 kg cm^−2^ for 25 min. After that the autoclave was stopped and waited until it cooled down. The medium divided into 30 beakers (25 mL each). Hormone was not added (control) to the first 10 beakers and BAP at 2.0 mg/l was added to the 11–20 beakers and NAA 0.2 mg/l was added to the rest 21–30 beakers, respectively.

#### 2.1.2 Plant materials

Pineapple crown was collected from farmer garden and placed in a beaker, washed thoroughly having water and sterilized with Clorox (20% for 25 min). The cutting slice were then rinsed twice in distilled water for 5 min and cultured in cylindrical glass jar [4] with a rimmed neck and plastic cover containing 25 mL of hormone free MS medium (10 jars), medium with BAP at 2.0 mg/l (10 jars) and NAA 0.2 mg/l (10 jars), respectively [Fig. 1]. The cultures were transferred to incubation room and kept under the constant temperature of 25 °C and photoperiod of 16 h of light provided by fluorescence lamp.

#### 2.1.3 Data collection

After 15 days callus started to initiate and after 60 days of incubation, data were collected. The number and length of shoots/explants and the total number of shoots produced were calculated and used for evaluation of the different treatments. The shoots removed from the cultures, weighed, separated into individual shoot for counting the number and measuring the length and weight of shoots.

#### 2.1.4 Total sugar determination

Total soluble sugar was determined according to the phenol-sulphuric method of [5].

#### 2.1.5 Determination of chlorophyll a and b

Total chlorophyll was determined according to the methods of [6]. The method consisted of repeated acetone extraction, until obtained colorless residue, with a pestle and mortar and filtered over filter paper (Whatman No.1 equivalent). The extracts were made up to 50 ml with acetone. The concentration of chlorophyll a at 666 mm and chlorophyll b at 653 nm was measured in a Shimadzu UV 160 A spectrophotometer. The amount of chlorophyll a and b was calculated according to the formula of [6], [7].

#### 2.1.6 Nutrient content determination

Nutrient content (NO_3_^--^ and K+) was determined by using Horiba Scientific NO_3_ and K meters (Japan). 3 drops of juice sample were put on the disc sensor of the meter using small dropper and data were displayed and recorded [8].

### 2.2 Experiment 2

#### 2.2.1 Plant materials

Inflorescence male bud of *Musa acuminata* cv. Pisang Mas were obtained from a farm Johore Bahru, Malaysia. Male inflorescences were collected when all the female flowers in a bunch were completely exposed and were cultured.

#### 2.2.2 Media preparation

MS semi solid medium (MS control) were prepared and MS supplemented with 10 mg/l, of N6-benzylaminopurine (BA), 1 mg/l of Indole-3-Acetic Acid (IAA), 2.0 mg/l gylcine, 0.4 mg/l (ppm) thiamine, HCl, 0.5 mg/l nicotinic acid, 0.5 mg/l pyridoxine, 10 mg/l ascorbic acid and 30 g/l sucrose [6]. Other media were supplemented accordingly with NAA 1 mg/l. The pH of the medium was adjusted to 5.8 prior to autoclaving and put into the autoclave having properties mentioned in Experiment 1. Thus media were prepared.

#### 2.2.3 Cultural procedure

The male inflorescence (bud) of banana were dissected and shortened to 6 cm in length. The explants were then disinfested with 70% volume of alcohol for 10 min and rinsed with sterile distilled water three times. Then 50 explants were cut longitudinally to make it half and were placed onto a semi solid medium (Fig. 2). The cultures were transferred to incubation room and kept under constant temperature of 25 °C and photoperiod of 16 h of light provided by fluorescence lamp.

#### 2.2.4 Data collection

After 21 days, explants were swelled up and turned green in color with the superficial bract curved outwards and exposing the rudimentary flowers. The white rudimentary flowers which appeared as white proliferating floral meristems were selected and cut into pieces and sub-cultured in the MS semi solid medium supplemented the same media and concentration of hormones mentioned previously. Finally, after 30 days of subculture, the individual callus and shoots were separated, washed and measured.

#### 2.2.5 Total sugar determination

Total soluble sugar was determined according to the experiment 1 which was mentioned above.

#### 2.2.6 Determination of chlorophyll a and b

Total chlorophyll was determined according to the experiment 1 which was mentioned above.

#### 2.2.7 Total phenols

The total phenolic content of explant was determined by using the Folin–Ciocalteu assay [9]. Folin–Ciocalteau (FC) colorimetry was based on a chemical reduction of the reagent, a mixture of tungsten and molybdenum oxides. 1 ml of leaf juice, gallic acid calibration standards and Folin–Ciocalteau (FC) reagent were stored in the dark and discarded if reagent had become visibly green, sodium carbonate solution (100-ml) was used in the volumetric flask. Spectrophotometer was set to 765 nm with 1-cm, 2-ml plastic or glass cuvettes. 1 ml of extract was added to 25 ml of volumetric flax, containing 9 ml of distilled water. A reagent blank was also prepared. 1 ml of Folin–Ciacalteu׳s phenol reagent was also added to the mixture. The solution was diluted with distilled water and mix and incubated at room temperature. Absorbance against reagent blank was determined at 750 nm with an UV–vis Spectrophotometer Lambda 5 and expressed as mg gallic acid equivalent. GAE/100 g fresh weight.

#### 2.2.8 Antioxidant as DPPH activity

The DPPH free-radical scavenging activity was determined as described in [10].

#### 2.2.9 Nutrient content determination

Nutrient content (NO_3_^--^ and K^+^) was determined according to the experiment 1 which was mentioned above [4].

#### 2.2.10 Design and statistical analysis

Randomized block design was used during sampling setting. Standard deviation and then standard error was made to compare the replicates. Least Significant Difference (LSD test) at *p*=0.05 was used for data analysis.

## Figures and Tables

**Table 1:** Shoot weight and stem length at different media from pineapple explants.

Medium	Shoot weight	Shoot number	Shoot length (cm)	Stem length (cm)	
BAP 2.0	1.66±0.05	22.2±0.1	9.1±0.07	6.1±0.05	
MS	1.41±0.04	16.4±0.2	6.9±0.05	5.2±0.05	
NAA 0.2	1.2±0.03	12.1±0.1	5.8±0.06	5.5±0.0	

Mean±SE (*n*=10).

**Table 2:** Total sugar, chlorophyll and nutrient content determination from pineapple explants.

Medium	Total sugar	Chlorophyll content	K^+^ content(ppm)	NO3^--^content (PPM)	
BAP 2.0	6.66a	4.2a	269a	385a	
MS	4.41b	3.4b	231c	344c	
NAA 0.2	5.2ab	3.1ab	245b	359b	

Same letters are not significant at the 5% level by LSD test.

**Table 3:** Callus weight, shoot number and length determination from banana explants.

Medium	Callus cell weight	Shoot number	Stem length (cm)		
BAP+IAA	3.2±0.04	12.2±0.06	2.1±0.02		
MS	1.8±0.01	5.4±0.07	1.3±0.02		
NAA	2.3±0.02	8.1±0.05	1.7±0.01		

Mean ±SE (*n*=10).

**Table 4:** Total sugar, chlorophyll, total phenol, DPPH activity and nutrient content determination.

Hormone and media	Total sugar	Chlorophyll (micro g/100 g)	Total phenol GAE/100 g	K^+^ content	NO3^--^ content	DPPH activity mg/100 g
BAP+IAA	3.2a	5.2a	421a	259a	355.1a	15.4a
MS	1.8b	3.4c	375c	236bc	334.5bc	10.0bc
NAA 10	2.3b	4.1b	388bc	244b	335.2b	12.6b

Same letters are not significant at the 5% level by LSD test.
