# Supplementary material for: Callus cell, shoot and stem proliferation data from pineapple crown and banana inflorescence in vitro: Biochemical and antioxidant properties
Source: Data Brief. 2016 Dec 6;10:340–5. doi: 10.1016/j.dib.2016.11.097 (PMC5157706; doi:10.1016/j.dib.2016.11.097)
Supplement: Supplementary file 1 — Supplementary material [file mmc1.docx]

**Conflict of interest**

Dear Editor

We do not have any conflict of interest in publishing the research work in the Data in Brief.

Best Regards

Dr. ABM sharif Hossain

(on behalf of all authors)
